# Diagnosis

**Published:** 1871-07

**Authors:** 


### DIAGNOSIS.

A visitation to any first-class surgical instrument manufactory—like Geo. Tiemann & Co.'s, New York—will astonish the non-professional visitor, when he has explained to him the numerous and varied instruments that have been devised to assist the physician in making examinations of his patients, in order to detect the various diseases to which they are subject. No matter what organ of the body is diseased, some instrument, or means for assisting the physician in his examination, will be shown him, so that the only wonder will be, how an intelligent physician can go estray in making up his diagnosis of a case.

We have the *laryngoscope*, by means of which the throat, air-passages, and speaking apparatus can be minutely examined, revealing the presence of tumors, obstructions, congestion, or ulceration. With the *endoscope* we are permitted to examine the urethra and interior of the bladder, where foreign substances, or any form of disease can be detected. Then, we have the *ophthalmoscope*, the most wonderful instrument recently placed in the hands of the oculist. It affords him a distinct view of the entire contents of the eye. By its use, the skillful observer can discover any abnormal change in the humors of the eye, can detect minute foreign substances that may have found lodgment there, is enabled to determine the presence of paralysis, congestion, or other diseased conditions of the retina, and can frequently detect diseases of the brain, and many other disorders, not of the eye, by certain evidences that can be recognized in the appearance of the retina.—And, now we are told that a physician of New Orleans has just completed an instrument, by the aid of which, and a powerful incandescent light, of an oxyhydrogen blow-pipe, or calcium light, he has succeeded in completely illuminating the abdomen and thorax, so as to plainly distinguish the entire internal machinery of these parts, enabling him to determine the presence of abnormal growths, or disease. Then, we have the *sphygmograph*, an ingenious instrument, recently invented, to detect diseases of the heart or arteries. It is applied to the wrist, in such a manner as to receive the pulsations of the radial artery, or "pulse," and accurately traces the pulse-wave upon a piece of paper. By the inspection of this tracing, the physician can determine the nature of the defective circulation, as to whether it is a functional or organic difficulty of the heart, and which of its parts are implicated in the trouble. It is also valuable in detecting many diseases, so that no practitioner of medicine, who is thoroughly "up" in his profession, will be found without a sphygmograph.

So numerous are the instruments for examination of various diseases, to be found in every well appointed physician's office, now-a-days, that we have not sufficient space in which to describe them. And, not only have we had great improvements in our means of diagnosis, but also in the manner of treating disease. Indeed, in no other science has there been such rapid progress as in the medical; which leads us to remark that we cannot agree with our talented contributor, Mr. B. H. PRATT, in the opinion which is expressed in his article in the present number of the BISTOURY, to the effect that physicians have made no progress in the management of disease since the days of our venerable grand-parents. We are fearful our friend has fallen into the hands of some "old fogy," who has plied him with calomel and jalap, for he certainly could not form such an opinion after having employed and conversed with an intelligent and reading phyisician of the present day. We have only to pick up one of the many excellent works on the practice of medicine, and on the various specialties, and compare it with a like treatise written fifty years ago; fifty years is a short time in which to work great changes in any of the established sciences, yet, will even the casual observer be able to distinguish wonderful discoveries, and marvelous improvements, in the management of diseases during a half century.

